# Illness-Related Domestic Violence Experiences of Individuals Diagnosed with Obsessive Compulsive Disorder: A Qualitative Study

**DOI:** 10.1192/j.eurpsy.2024.1311

**Published:** 2024-08-27

**Authors:** Z. Bayram, M. C. Aktaş, H. Gundogdu

**Affiliations:** Psyhiatric and Mental Health Nursing, Van Yüzüncü Yıl University, Van, Türkiye

## Abstract

**Introduction:**

Obsessions and compulsions often focus on routine life actions such as consuming, bathing and living in public, so the disorder interferes with the social, family and occupational functioning of both the individual and their caregiver. In OCD, caregivers are often involved in the ritual behaviors of the individual, either by providing avoidance or by assisting in ritual behaviors. Individuals with OCD are often exposed to aggression from family members because of these behaviors. Research has shown that individuals with OCD may experience illness-related domestic violence. In the literature review on the subject, there were no qualitative studies examining the experiences of individuals diagnosed with OCD towards domestic violence caused by the symptoms of the disease. A mix-method study conducted by aimed to explore the illness-related domestic violence experiences of individuals diagnosed with OCD. The study utilized both quantitative and qualitative methods to gather data from individuals with OCD. The findings of the study revealed that a significant number of individuals with OCD reported experiencing domestic violence related to their illness. This study highlights the importance of addressing the issue of domestic violence in individuals with OCD and the need for appropriate interventions and support.

**Objectives:**

This study was planned to examine the experiences of individuals diagnosed with OCD regarding domestic violence caused by the symptoms of the disease in Turkey.

**Methods:**

The sample of the study was determined by purposive sampling method. The study was conducted with individuals diagnosed with OCD who were being treated in the psychiatric clinics of two university hospitals. The study used mixed methods and was planned in two phases. In the first stage, Socio-Demographic Data Form and Yale-Brown Obsession Compulsion Scale were used. After reviewing the results of these scales, individual in-depth interviews were conducted with 20 patients who had a history of domestic violence and scored above 16 on the Yale Brown Obsessions and Compulsions Scale. Data saturation was deemed to have been reached and data collection was terminated.

**Results:**

The analysis of the data is still ongoing in detail by the researchers. The findings and relational implications of the study will be presented.

**Conclusions:**

It is thought that the results of the study will provide a basis for further research and intervention programs and contribute to the literature by determining the types of violence experienced by individuals diagnosed with OCD from family members due to the disease, how patients feel in the face of the problems they experience and how they cope with it. In addition, the results of the study are expected to help intervention programs to be developed for families to strengthen treatment adherence.

**Disclosure of Interest:**

None Declared

